# Individual sustainability competence development in engineering education: Community interaction open-source learning

**DOI:** 10.1371/journal.pone.0294421

**Published:** 2023-11-28

**Authors:** Hangyang Li, Zhiliang Huang, Tongguang Yang, Wenfang Zhang, Mingjie Chen, Zehai Li, Ke Ren

**Affiliations:** Smart City Energy Perception and Low Carbon Manufacturing Science Popularization Education Base of Hunan Province, Hunan City University, Yiyang, Hunan, China; Massachusetts Institute of Technology School of Engineering, UNITED STATES

## Abstract

This empirical research creates and assesses a community interaction open-source learning framework. The framework established an efficient open-source learning environment for engineering courses to develop undergraduates’ sustainability competencies. The teaching practice of the framework was designed into three stages: course preparation, theory lecture, and project practice. In the teaching practice, community interaction elements were embedded, including community/student two-way selection, systemic teaching and difficulty discussion, expert/student negotiation on teaching forms, teacher/expert coordination on teaching contents, and expert/student two-way feedback on schedules. The interaction elements between students, teachers, and experts enhanced the effectiveness of open-source learning in engineering courses. The experimental results showed that the students exhibited a positive attitude and high participation in the learning procedure and reported a sense of achievement in the project practice. The open-source learning framework significantly improved systemic thinking, conceptual understanding, interdisciplinary collaboration and professional skills. It enhanced students’ key sustainability competencies and laid the foundation for them to become expertise-based innovators and open-source community contributors.

## 1 Introduction

The education community attempts to enhance students’ individual sustainability competence to meet real-world challenges through developing a series of problem-driven and outcome-oriented educational theories and teaching approaches [1]. Existing studies have reached a basic consensus on the definition of sustainability competencies, including systemic thinking, interdisciplinary collaboration, and professional skills [2, 3]. The key competencies are expected to support students to become problem solvers, change agents and transition managers in the future [4]. Therefore, the professional curriculum design should build competence-oriented learning environments and develop efficient teaching approaches for sustainability competence development. An open-source learning environment has characteristics such as innovation, openness, freedom, sharing, and collaboration, coinciding with the goals and features of sustainability education [5]. The use of open-source technology or projects in professional courses has a long history. Early open-source education primarily taught the use of open-source software or project development skills based on the software [6]. With the development of the open-source community and internet technology, the culture and concepts of open-source have gradually entered the classroom [7]. Besides, vocational education performed by non-university organizations is also booming, and the massive open online course (MOOC) gradually emerged and improved [8]. Consequently, the open-source learning model has attracted extensive attention from educational and academic communities, and is increasingly valued by higher education and vocational education.

The open-source learning models in university engineering education can be roughly divided into two categories. The first category is open-source development courses, which guide students to use a software platform to build an open-source community or build management rules for achieving efficient community governance. It does not explore and discuss open-source technologies in depth, making it unsuitable for cultivating professional skills for engineering students, such as mechanical, electrical, and civil engineering. The second category is professional courses, which use open-source technologies or projects to perform professional course teaching [9]. It guides students to solve practical engineering problems using technologies that contain massive open resources. It is conducive to developing individual sustainability competence, such as systemic thinking, interdisciplinary collaboration, and professional skills [10].

However, the practice of open-source learning models encounters challenges in the learning environment and teaching approach. Firstly, the open-source projects were developed by professional teachers or experts selected from communities. Due to the lack of participation and prior knowledge, students may have insufficient learning motivation or cognitive impairment. Secondly, even if the students were allowed to choose their project topics, there might be a lack of professional instructors for the topics, thus making it difficult for them to receive timely feedback and achieve learning effects within a limited schedule. Thirdly, real-world challenges usually involve multidisciplinary knowledge. It is difficult for professional teachers to integrate interdisciplinary knowledge and more challenging to guide students to perform engineering practices under an interdisciplinary framework.

This study explores how to embed the interaction between teachers, students and open-source communities, thus supporting the key sustainability competence development of engineering undergraduates. We propose a general open-source learning framework applicable to teaching engineering courses and test the learning effect from a corresponding teaching approach. The framework aims to make students become willing, capable, and rule-compliant project practitioners and community contributors. This study follows an empirical research paradigm and addresses the challenges of implementing open-source learning in engineering courses. The research questions of this paper are as follows.

1. How to build a community interaction open-source learning framework to support engineering course teaching?
2. How do participants view incorporating an open-source learning framework in engineering courses?
3. How does the open-source learning framework strengthen participants’ sustainability competencies?

The rest of this paper is structured as follows. Section 2 provides a literature review on sustainability competence development and open-source learning models. Section 3 proposes a community interactive open-source learning framework and explores its practice path in professional courses. Section 4 gives the data analysis on key competence development. Section 5 answers the research questions. Section 6 illustrates the research limitations. Section 7 concludes and discusses future research directions.

## 2 Background

### 2.1 Individual sustainability competency development

Corporate sustainability faces various challenges, and each issue should be analyzed and solved within a specific context and agenda. Practitioners develop innovative solutions based on individual and teamwork competencies to address complex sustainability issues. The competencies of technical specialists and engineering managers are critical to corporate sustainability. They are called problem solvers, change agents and transition managers. As problem solvers, they need to undertake four core tasks, including orientation, reaching common ground, performing pilot projects, and embedding results [2]. Individual competencies play a crucial role in acting as change agents when addressing path dependence and complex settings in sustainability [11].

Communicating effectively with different decision-making domains to propose systemic sustainability solutions is difficult. It requires a highly qualified transition manager team [12]. Key competencies in sustainability summarized as systemic thinking, interdisciplinary collaboration, and expertise-based action [13, 14]. Systemic thinking is critical to understanding complex system properties, e.g., interactions between components, emerging phenomena, and network organization [15]. The systemic thinking-oriented teaching approach is structured by context-driven enquiry, supported by learning power, positioned at the interface of knowledge generation and use, and grounded in a commitment to sustainable development [16]. Interdisciplinary collaboration is becoming more widespread thanks to its importance in increasing creativity, innovation, and team performance. Interfusing interdisciplinary collaboration with professional education can cultivate learners’ integration and creativity and enrich practical experience [17]. Expertise-based action results from a deep and rich knowledge base from extensive experience in a field. The key to developing competence lies in well-organized knowledge structure, processing styles, practical experience, availability of feedback and performance under pressure [18].

The above literature suggests that sustainability competency development places a demand on the learning environment [16–18]. They include the diversity of teaching materials, teacher’s knowledge structure, students’ engagement, instant feedback and teamwork. Engineering courses are limited by schedule, cost, and organization, posing challenges to developing students’ sustainability competencies. Therefore, it is essential to explore an effective learning environment for sustainability competency development, bridging the gap between the high-quality talent demands and engineering education.

### 2.2 Open source learning environment

Open-source learning environments aim to make learning more accessible and equitable, serving as a vehicle for educational change [19]. It is defined as open-source digital resources for teachers and students to study, teach, and learn. Generally, the resources are integrated into a digital platform, including content, development, use and distribution. Open licenses were granted to users based on specific rules [20]. In higher education, an open-source learning environment typically consists of open-source learning tools, online courses and tutorials, accompanying courseware and materials, and reference e-books and e-journals. Compared to conventional MOOCs, the learning environment focuses on providing open-source learning tools of software and hardware [21]. Open-source software licenses users to modify and distribute its source code. The development of many projects follows the open-source concept and has gained significant influence worldwide [22], e.g., the Linux operating system and the Apache web server.

Moreover, open-source software inspired and promoted another trend. It introduced the openness concept into electronic hardware design, thus forming open-source hardware [23], such as the Arduino platform and OpenPilot system. Open-source hardware maximizes individual abilities to make and use hardware using readily available materials, components, standard procedures, open platforms, available content, and design tools.

Open-source tools are well-suited for various educational purposes [24]. They help students use novel technologies more fluently and expressively, enabling them to explore abstract concepts in science, mathematics, and engineering [25]. It can provide a better learning experience and encourage students to actively participate in design and innovation [26]. Existing studies demonstrated the advantages of open-source learning environments in promoting conceptual understanding, professional skills, systemic thinking, and interdisciplinary collaboration [24–26]. However, there are challenges to implementing the open-source learning model in engineering courses. Proposing efficient teaching approaches and performing empirical studies will help to bridge this gap.

### 2.3 Stakeholder needs for open source learning

Students, teachers, universities, open-source communities, and businesses are the five stakeholders of open-source learning implementation in engineering education [27]. The openness concept can enhance students’ interest and recognition of open-source technologies and cultivate their desire for innovation. An open-source learning tool contains high-quality program codes and design ideas, and open-source development is one of the current mainstream innovation models. Engineering students must learn and master it [28]. Open-source practical teaching usually involves multiple disciplines and research directions, and it is difficult for professional teachers to maintain a cutting-edge state in the directions. Therefore, it is a daunting task to guide students in innovative practice. The open-source learning model is expected to significantly reduce the difficulty for teachers to engage in practical teaching [29]. Due to the rapid iteration of internet-driven technological innovation, using conventional methods to establish time-sensitive valuable teaching resources has brought heavy management and economic burdens to colleges and universities. Open and shared teaching tools alleviate the dilemma of practical teaching in engineering education [30].

High-quality contributors are the basis for promoting the sustainability of open-source communities, and engineering students are potential contributors. They participate in open-source project development through innovative practices to enrich community resources [31]. There seemingly exists a gap between engineering education and corporate sustainability. Higher education focuses on constructing knowledge systems, while enterprises need talents with sustainability competencies.

Open-source learning allows students to get in touch with actual product development in advance. It has a more flexible schedule than traditional corporate internships, reducing the costs of identifying and cultivating potential practitioners [32]. The stakeholders have agreed on a strong need for open-source learning. However, proposing a general open-source learning framework to form a long-term mechanism that meets the stakeholder needs seems to be an urgent issue to be solved.

## 3 Materials and methods

### 3.1 Community interaction open source learning framework

The community interaction open-source learning framework aims to make students become willing, capable, and rule-compliant project practitioners and community contributors. The framework introduces open-source technologies, community operation and governance rules to students. It enables them to understand the significance of contributing to individual and community sustainability, inspiring the internal drive for active learning. Students are guided to participate in practical projects to master applying open-source technologies to solve engineering problems, enhancing systemic thinking and professional skills. Under the constraints of community rules, students and community teams use open-source tools orderly and contribute self-developed open-source projects to the community based on design projects in professional courses. Thus, it cultivates students’ interdisciplinary collaboration and action. The open-source learning framework adopts a teaching strategy of cooperation between professional teachers and community experts, giving full play to the advantages of primary theory teaching and open-source technology practice.

The open-source learning framework consists of theory lectures and project practices. The lecture contents include concepts of open-source culture, community framework, project evaluation, and open-source licenses. systemic theory teaching enables students to master the knowledge and skills of numerical simulation, laying the foundation for them to become engineers. Project practice is guided by community expert teams.

**Fig 1:**
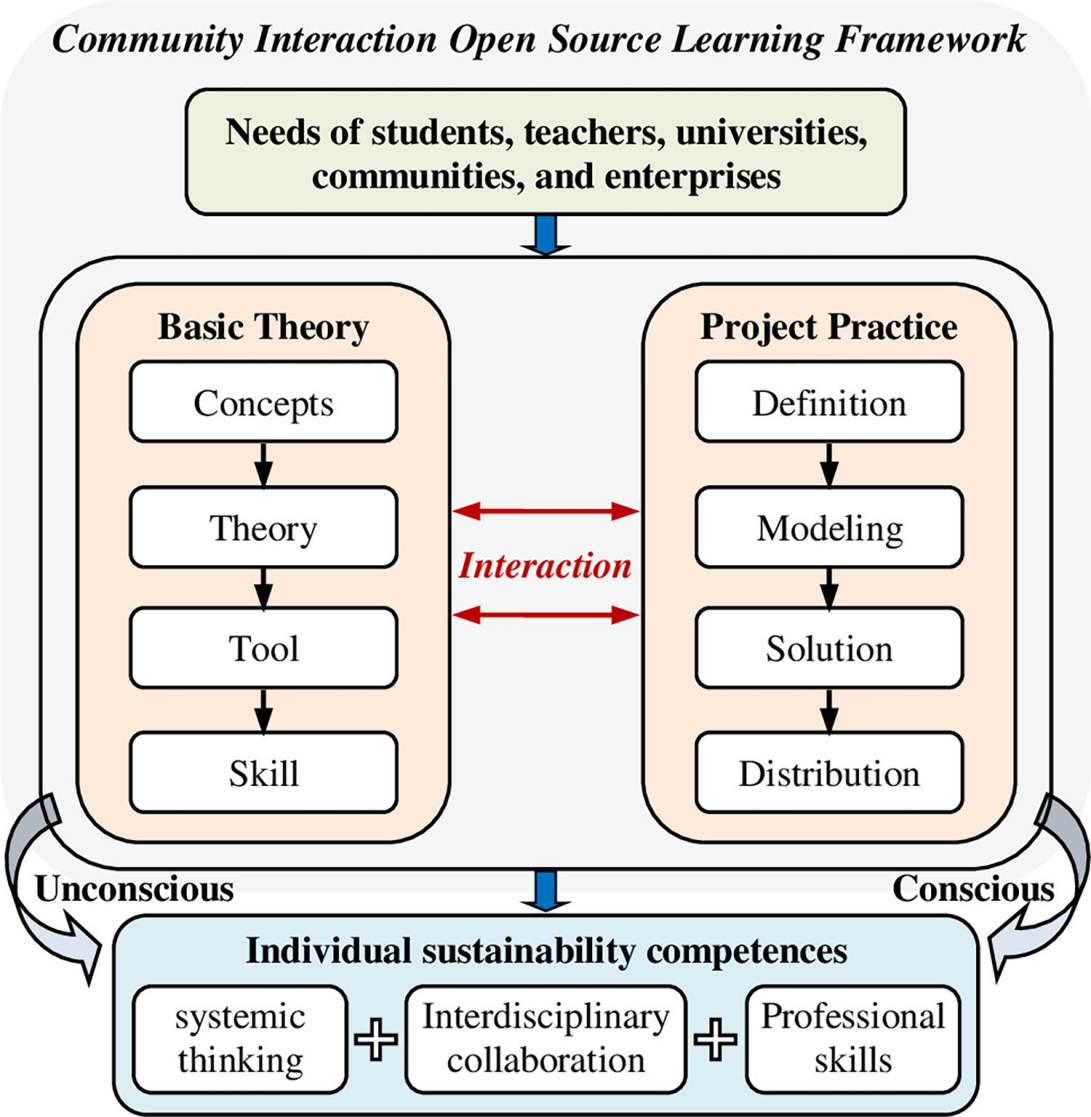
The community interaction open source learning framework.

The teachers provide project topics that meet the course teaching objectives. The students choose topics based on their personal study interests and community teaching resources. According to the classification of community research directions, the students establish connections with the community experts, who will provide relevant open learning resources and practical guidance. The guidance steps include background knowledge, problem definition, modelling based on the open-source tool, cloud platform solution, and simulation APP production. The project practices encourage students to use the open-source tools to solve engineering problems. Thus, it enhances students’ sustainability competencies and guides them to contribute open-source projects to the community. The proposed community interaction open-source learning framework is shown in Fig 1.

### 3.2 Courses and participants

The empirical research was part of the *Industry-University Cooperation Collaborative Education Project*, initiated by the Higher Education Authority. This project encouraged cooperation between universities and enterprises to explore the reconstruction of the engineering curriculum system. A course of *Structural Finite Element Analysis* is adopted to conduct the empirical research. It is a professional course for engineering undergraduates in mechanical, electrical, civil, and materials majors. The course aims to foster numerical simulation skills in mechanical/thermal/electrical physical fields of engineering structures under complex conditions. The course employed project-based learning to guide students in solving engineering problems using simulation techniques. In the actual classroom, the empirical study on the community interaction open-source learning framework was carried out.

A total of 387 participants were third-year undergraduates from ten teaching classes. Six classes were majoring in mechanical engineering, with 202 students (52.2%). And four classes were majoring in electrical majors, with 185 students (48.8%). The participants comprised 322 males (83.2%) and 65 females (16.8%). The mean age of the participants was 20.5 years, with a standard deviation of 0.59 years. The instructors included sixteen professional teachers and five community teams. Each professional teacher was a doctor in electrical or mechanical engineering, with an average of 6.5 years in teaching experience. Community experts were proficient in multi-physics simulation and engaged in the performance evaluation and innovative design of engineering structures, electro-mechanical equipments, and electronic components.

### 3.3 Ethics statement

Before the teaching practice was carried out, the research purpose and plan were reviewed and approved by the academic ethics committee of Hunan City University. All participants have been informed of the research content and procedure, and were required to sign an informed consent form. We have obtained the written informed consent from all participants.

### 3.4 Teaching materials

Open-source learning materials were authorized by the open-source community (www.simapps.com), including a simulation platform, online courses, tutorials, project examples, and APP evaluation standards. The open-source community was built and operated by IBE Co., Ltd, which develops a general multiphysics simulation platform of Simdroid. The platform can provide numerical simulation solutions in industries of electronics, electric power, aerospace, and vehicles. The open-source education based on the platform was part of the University-Industry Cooperation Education Project (UICEP), initiated by the higher education authority.

The courses, tutorials, and examples were jointly developed by professional teachers and industry experts participating in UICEP. The course was called *Computer Aided Engineering Training Camp*, including the demonstration and teaching of structured simulation procedures, such as geometric modelling, meshing, boundary condition setting, solution, and result presentation. The tutorials were simulation applications for professional courses, such as electromagnetism, mechanics, heat transfer, and polymer materials. The examples consisted of thousands of simulation solutions corresponding to engineering problems in various industries. The APP evaluation standard was used to evaluate whether the open-source projects developed by learners met the publishing standards and was one of the criteria for measuring the learning effect. The learning materials are accessible online through the open-source community (https://www.simapps.com/).

### 3.5 Teaching procedure

**Fig 2:**
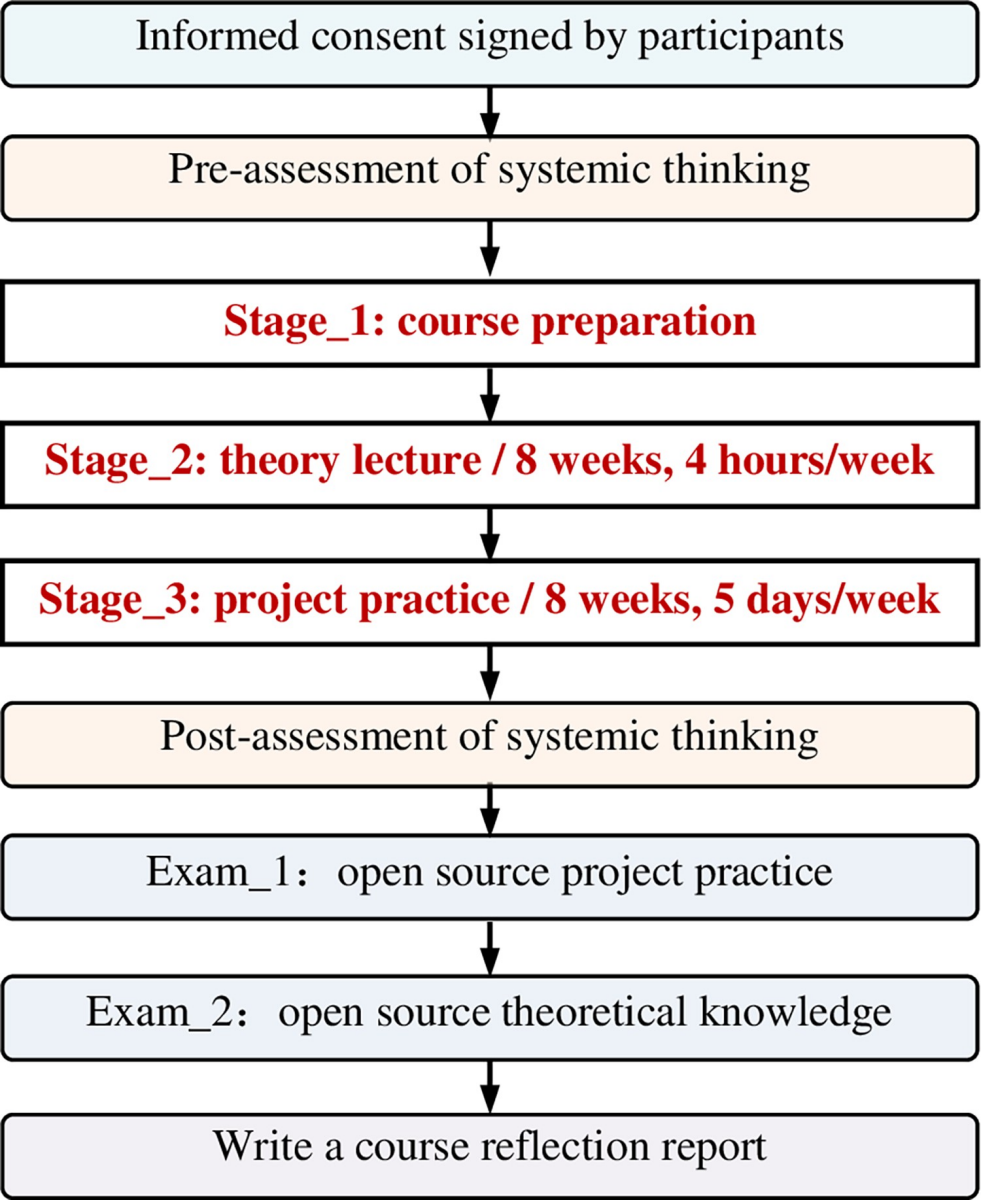
The implementation procedure of the empirical study.

A community interaction open-source learning framework was applied to real classrooms for empirical study. The teaching practice consisted of three primary stages: course preparation, theory lecture, and project practice (Fig 2). The participants were assessed on systemic thinking before and after the courses. After the theory lecture, the participants selected topics for project practice. The professional skill exams and course reflection were arranged at the project practice end.

### (1) Stage_1: Course preparation

The professional teachers and community experts discussed and determined the project topics. The topic selection took into account the appeals of stakeholders in open-source learning, including practice difficulty (students, teachers), teaching objectives (university), project contributions (community), and engineering application (enterprises). To stimulate learning interest, the topics were classified according to application industries, including electronic packaging, electrical equipment, vehicles, machine tools, mechanical arms, and robots. Each topic corresponded to a task description, providing learning objectives, design content, outcome requirements, schedule and references.

A practice teaching template was provided to the community teams for standardizing the teaching contents and procedures of project practices. The template stipulated the necessary teaching procedures, including the introduction of background knowledge, setting of preview content, and grading of basic/advanced tasks. Some optional approaches were suggested for the community team. For example, the practical guidance can be performed online or offline, determined through consultation between experts and students. Enterprises are important clients of the open-source community. It is encouraged that community experts bring in corporate resources and combine self-developed tools and resources to assist guidance.

The two-way selection mechanism of community and students dealt with the risk of unbalanced guidance burdens of community teams. Before project practices, information such as task description, corresponding community team, and expected number of mentors was released to all students. Each student applied to the desired community team (no more than three). The community team responded to applications based on the student’s past grades and description of interests. The mechanism provided the students and community with the opportunity to choose freely. And it balanced the guidance burdens of community teams through adjustment.

### (2) Stage_2: Theory lecture

The theory lecture was undertaken by the teachers, and the teaching form primarily relied on classroom lectures. The theory lectures started with introducing open-source technologies and communities, focusing on the finite element simulation theory and open-source simulation tools. The stage was limited to 32 hours over eight weeks at four hours per week. The lecture contents were organized around the following modules: ① the introduction to open-source technology, e.g., open-source culture, community framework, project evaluation, and license; ② the finite element simulation theory, including the elastic mechanics equations, the stiffness matrix of plane problems, the solution of nonlinear problems, and the discussion of technical difficulties; ③ the execution of complex simulation steps, such as geometric modelling and meshing; and ④ the critical points in different types of problems, e.g., statics, dynamics, structural nonlinearity, and particular analysis types. The lecture content and schedule are listed in Table 1.

**Table 1:** The content and schedule of theoretical teaching.

Content	Description	Hour	Week
Open source introduction	Concept and culture, community framework and system, simulation APP evaluation, and license.	2	Week_1
Simulation theory	Basic equations of elasticity, stiffness matrix of planar problems, solution of nonlinear problems, and discussion of technical difficulties.	2	Week_2
Geometric modelling	Structural feature simplification, structural symmetry handling, assembly modelling, and geometric model import.	2	Week_3
Meshing	Element selection, mesh size setting, situation encryption techniques, and mesh distortion handling.	2	Week_4
Static analysis	Analysis procedures such as cantilever beam bending, plate stretching with holes, shell deformation with fixed supports on sides, and bar torsion.	2	Week_5
Kinetic analysis	Analysis procedures such as free vibration of rectangular plate, cantilever beam mode, crank slider mechanism motion, crankshaft harmonic response.	2	Week_6
Structural nonlinear analysis	Analysis procedures such as bolt contact with preload, multi-step loading of pipe clamps, sealing performance of rubber gaskets, and large deflection bending of rectangular plates	2	Week_7
Special analysis types	Analysis procedures such as L-type beam buckling, heat sink temperature field, circuit board thermal coupling, and slender rod instability	2	Week_8

### (3) STAGE_3: Project practice

The practice of open-source projects for students is mainly guided by the community team. The guidance adopted a combination of online and offline teaching. The stage lasts for two weeks, five working days per week. After the practice, each student made ten simulation APPs. The APPs can be based on one open-source project, or multiple similar projects.

The professional teachers were responsible for coordinating the differences in the guidance of various community teams and bridging the communication gap between students and experts in interdisciplinary collaboration. Each team carried out structured instruction according to the practical teaching template. By combining industrial resources and mentoring expertise, a team usually develops a unique style of guidance. Therefore, it is necessary to coordinate the guidance content of multiple teams to ensure systematization and rigour. The teams may focus too much on technologies relevant to the current project in their guidance. As a result, a general introduction to open-source practice may be missing, or it may overlap with theoretical teaching. Also, there may be bias towards commercialization and publicity. Thus, the focus of inter-team coordination should be to clarify the general education goals in the open-source practice. Based on the project development experience of the community teams and considering the acceptance of students, the open-source projects should be introduced from the perspective of knowledge application. Moreover, attention should be paid to the organization of the knowledge system, not just detailed descriptions for specific projects.

A two-way feedback mechanism was constructed to facilitate interdisciplinary collaboration. Community experts know less about the psychological and learning status of the participants. In practical guidance, it is easy to have poor communication, affecting the guidance effectiveness. Therefore, the teachers were a bridge between the experts and students. They followed up the whole procedure and collected two-way feedback in time. On the one hand, the professional teachers communicated with various community teams to track and guide progress. On the other hand, they communicated with students promptly to obtain feedback, providing a basis for experts to adjust the instruction schedule. In the two-way feedback, the teachers and experts guided students to communicate efficiently in interdisciplinary collaboration and enhance their teamwork and open collaboration skills.

**Fig 3:**
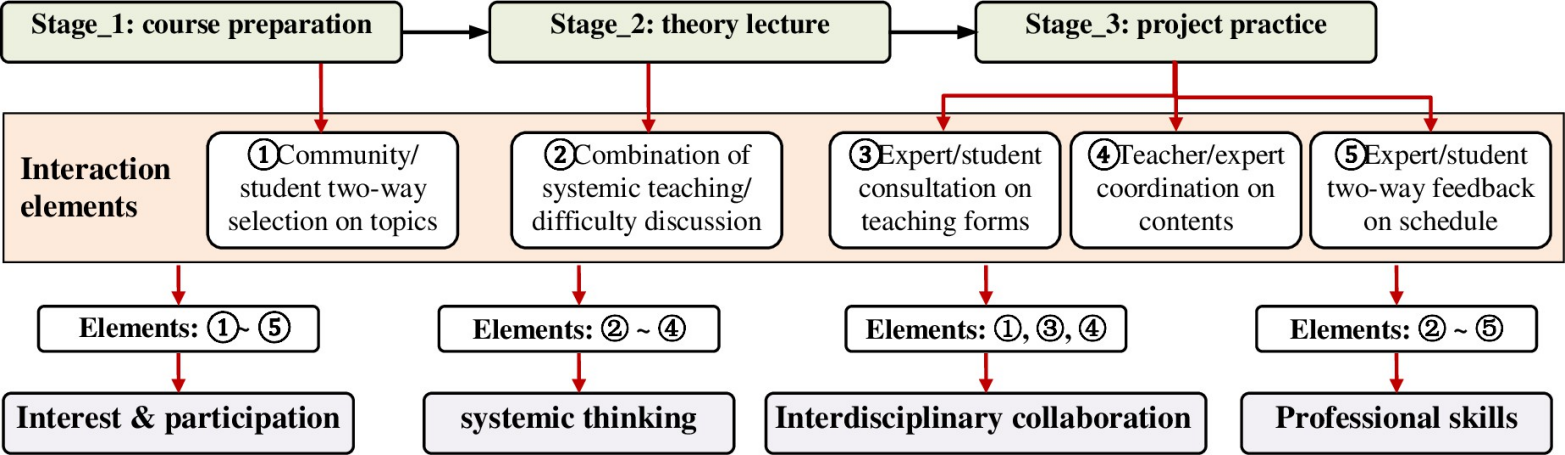
The relationship between teaching stages, interaction elements, and possible contributions.

To sum up, the practice of the proposed framework included three stages of course preparation, theoretical teaching, and project practice. The core difference from conventional open-source learning lay in the implantation of community interaction elements. The elements aimed to strengthen open-source learning effectiveness, learning interest land participation, and promote students’ sustainability competencies, such as systemic thinking, interdisciplinary collaboration, and professional skills. The relationship between teaching stages, interaction elements and their possible contributions is shown in Fig 3.

### 3.6 Data collection

This study employed systemic thinking assessments, professional skill exams, and course reflections to collect qualitative and quantitative data. The participants’ systemic thinking was assessed before and after the course experiment, and the results were considered pre- and post-assessment data. The professional skill exams were organized by the higher education authority, and students from colleges and universities participated in the exams. The pass rates in similar universities and majors with different teaching approaches were used to compare the learning effect of the experimental group. At the course end, each participant submitted a reflection report for analyzing their perceptions of the open-source learning framework.

### 3.6.1 Systemic thinking assessment

Systemic thinking is the ability to recognize and synthesize patterns, interactions and dependencies in a series of activities. The systemic thinking assessments were performed through a self-report scale. It was modified from the Systemic Thinking Scale with twenty items developed by Dolansky et al. [33]. The internal consistency testing using Cronbach’s alpha showed a coefficient of 0.89, verifying the scale validity. The scale contains twenty items, that is easy to administer and takes less than ten minutes to complete. Considering the context of open-source learning, we specified the general expressions in the original scale, such as organization, system, and environment. The participants described their thought processes in five closed options for each item. For example, the participant made choices among the options of Never, Seldom, Some of the time, Often, Most of the time to describe the thought process for "I keep in mind that proposed changes can affect the whole system". The response to each item was scored on a five-point scale, and the average score for all items was used to measure the participants’ systemic thinking.

#### 3.6.2 Professional skill exams

The professional skill exams included practical and theoretical exams. The practical exam required submitting one complete open-source project, including ten simulation APPs, one set of open-source codes and technical documentation. The community experts blindly reviewed the open-source projects to determine if the participant qualified for the theory exam. For disqualified projects, the participants have one opportunity to resubmit a revised version based on experts’ comments. The theory exam covered the knowledge points in simulation theory and procedures, including one hundred multiple-option questions. For each question, one point was awarded for a correct answer and zero for a missed or wrong answer. The participants took the centralized online exams. The answer sheets were submitted by the participants to the examination system, and the system automatically graded the sheets and gave the scores immediately. Participants who pass the professional skill exams can obtain the vocational-technical certificate of "Simulation Application Engineer (Junior)" issued by the education authority.

#### 3.6.3 Course reflection

The participants submitted an 800-word course reflection describing their open-source learning experience at the end. A structured template was provided, including learned simulation methods and tools, the most useful or attractive links, challenges and solutions, and a willingness to be a long-term contributor to the open-source community. Analyzing the reflection reports, we identified participants’ perceptions of the open-source learning environment and the relationship between participant competence improvements and the courses implemented. The teacher conducted a semantic analysis of the reflection reports.

The coding themes were discussed and determined by the sixteen teachers in response to research questions, as listed in Table 2. The template of the reflection report was designed based on the coding themes. The themes covered the teaching materials, course setting, and learning environment. Each theme included three sub-themes, pointing to the essential elements affecting the learning experience. For example, if a participant reported a positive attitude towards the *Structured simulation process demonstration*, the first sub-theme was encoded as 1, otherwise 0. The sum of sub-theme codes reflected the participant’s learning experience.

**Table 2:** The coding themes and sub-themes.

Theme	Sub-theme
Teaching materials	Structured simulation process demonstration
	Tutorials for professional courses
	Examples of actual engineering projects
Course settings	Course preparation stage
	Theory lecture stage
	Project practice stage
Learning environment	Teaching schedule adjustment
	Peer support from the community
	Sense of achievement in project practice

### 3.7 Data analysis

This research performed pre- and post-assessments for participants’ systemic thinking. The T-test was employed to analyze the difference between the two assessments. The difference was evaluated by the P-value and the effect size of Cohen’s d. P-value<0.05 indicates that the difference is statistically significant. Cohen's d≥0.8 means a big effect size of difference [34]. Besides, the Pearson correlation coefficient, i.e., *γ*∈[−1,1], was employed to measure whether the teacher’s experience affected the participants’ improvement under their guidance. If |*γ*|≥0.7, a strong correlation is supported [35].

The students submitted course reflection reports. Each report was coded by a professional teacher and a community expert to ensure inner-coder reliability. Cohen’s Kappa (*κ_c_*) was employed to measure the consistency between the preliminary encoding results and *κ_c_*≥0.6 indicates that the consistency meets the requirements [36]. If the consistency was satisfied, the teacher and expert discussed until consensus was reached on all differences.

## 4 Results

### 4.1 Systemic thinking assessment results

The systemic thinking assessment results are listed in Table 3. The participants’ mean score improved from 2.83 in the pre-assessment to 3.46 in the post-assessment, an improvement of 22.3%. A significant effect size emerged. The comparisons showed that participants’ systemic thinking was improved significantly by open-source learning. The participants were students majoring in mechanical and electrical engineering. Considering possible differences among different majors, all participants were further divided into two experimental groups: mechanical and electrical groups. Data analysis was performed for the two groups, and the results were similar to the global analysis. The systemic thinking of the mechanical and electrical groups increased by 22.4% and 22.1%, respectively.

**Table 3:** The comparison of systemic thinking assessment results.

Group	Mean scores of pre-assessment	Mean scores of post-assessment	Increase (%)	P-value	Effect size (Cohen’s d)
Overall	2.83	3.46	22.3%	<0.05	2.38
Mechanical	2.77	3.39	22.4%	<0.05	2.30
Electrical	2.89	3.53	22.1%	<0.05	2.47

Besides, the possible influences of participants’ gender and teachers’ experience on systemic thinking improvement were analyzed. The participants were divided into the male and female groups to compare the improvement. The results showed no difference in improvement between the two groups (P-value = 0.12). The correlation coefficient between teacher experience and participant improvement is *γ* = 0.071, indicating no significant correlation.

### 4.2 Professional skill exam results

The professional skills exams were divided into two links: practical and theoretical exams. Each participant obtained the theoretical exam qualification through the practical exam, and received vocational certificates through the theoretical exam. Three pass rates were considered to measure participants’ professional skills: practical, theoretical, and comprehensive. Two control groups were established with the exam participants from two similar universities and majors: Group_A and Group_B. Similar to the university of the experimental group, the two institutions were application-oriented universities. And the economic levels of the regions where they were located were comparable. The control groups came from mechanical and electrical majors, and the number and proportion of them were roughly the same as those of the experimental group. Group A and Group_B also used the Simdroid open-source simulation platform for project-based learning, but did not use the community interaction open-source learning framework.

Table 4 lists the professional skills exam results. The three pass rates of the experimental group were significantly higher than those of the two control groups. The practice, theory, and comprehensive pass rates of the experimental group were 85.0%, 75.2%, and 75.2%, respectively. Compared with Group_A, the pass rate advantages are 13.0%, 65.0%, and 65.0%. Compared with Group_B, the advantages are 10.3%, 55.5%, and 55.5%. It suggested that the community interaction open-source learning framework has an obvious advantage over conventional open-source learning.

**Table 4:** The comparison of professional skill exam results.

Results	Practice examination			Theory examination			Practice/Theory Synthesis		
	Experimental group	Control group_A	Control group_B	Experimental group	Control group_A	Control group_B	Experimental group	Control group_A	Control group_B
Pass-rate	85.0%	75.4%	77.1%	75.2%	45.6%	48.4%	75.2%	45.6%	48.4%
Advantage to Group_A	13.0%	/	/	65.0%	/	/	65.0%	/	/
Advantage to Group_B	10.3%	/	/	55.5%	/	/	55.5%	/	/

### 4.3 Course reflection results

Two preliminary encoding results were tested for consistency, and the *κ_c_* values and differences on each sub-theme are listed in Table 5. *κ_c_*>0.8 indicates that the preliminary results had high consistency. Thus the encoding reliability was verified. After a discussion between the two coders, the differences were eliminated, and the final result was formed. The data is counted from two dimensions, i.e., sub-theme and participant.

**Table 5:** The results of the reflection and statement examples.

Theme	Sub-themes	Cohen’s Kappa (*κ_c_*)	Number (%)	Examples extracted from the reflections
Teaching materials	Structured simulation process demonstration	0.93	253 (65.4%)	The demonstration of the simulation procedure in the online course guided me step by step through the thermal analysis of the circuit board.
	Tutorials for professional courses	0.92	269 (69.5%)	I found reference examples from *Materials Mechanics Simulation Tutorial*, thus overcoming the challenges of engine crankshaft structure simulation.
	Examples of actual engineering projects	0.88	316 (81.7%)	The engineering examples are interesting, especially for batteries and power semiconductor chips.
Course settings	Course preparation stage	0.96	156 (40.3%)	I successfully joined the community team for electromagnetic analysis of power systems, and I thank them for accepting me.
	Theory lecture stage	0.97	135 (34.9%)	The geometric modelling and meshing of the gearbox is difficult. But after reviewing the relevant theories and techniques, the difficulties have been resolved.
	Project practice stage	0.84	332 (85.8%)	I felt that it was similar to a conventional design, and it was enough to address the LCA analysis into a general performance function.
Learning environment	Teaching schedule adjustment	0.96	198 (51.2%)	I’m a bit behind with the progress of the dynamics simulation. After giving feedback to the teacher, the expert slowed down the progress.
	Peer support from the community	0.92	296 (76.5%)	There are many peers active in the community discussion boards, and they provided me with ideas to address the challenges of project practice.
	Sense of achievement in project practice	0.94	261 (67.4%)	My lithium battery thermal simulation APP was received and released by the community. I want to include this experience in my resume.

On the theme dimension, the proportion of participants with a positive view for each sub-theme was counted. 372 (96.1%) participants reported improvements in their course reflection reports. The improvements were related to the themes of teaching material, course setting, and learning environment. In terms of teaching materials, 253 (65.4%) participants said the structured simulation procedure demonstrations, 269 (69.5%) participants said the benefit of tutorials in understanding abstract concepts, and 316 (81.7%) participants reported interest in practical engineering simulation solutions. In course settings, the number of participants reporting improvements related to three course stages was 156 (40.3%), 135 (34.9%), and 332 (85.8%), respectively. In terms of learning environment, 198 (51.2%) participants highlighted the help of community experts to adjust the guidance schedule promptly, 296 (76.5%) participants mentioned peer support from the open-source community, and 261 (67.4%) participants reported a sense of achievement from contributing to the community. Table 5 presents the statistical results of course reflection and examples extracted from participants’ statements.

For each participant, the sum of sub-theme codes was counted as a reflection index, which reflected his learning experience. Theoretically, the highest value of the index was 9, and the lowest value was 0. The index of 9 showed that the participant holds a positive view on the nine sub-themes, i.e., an excellent learning experience. The average value of the indexes is 5.7, and the standard deviation is 1.6.

### 4.4 Correlation analysis

The Pearson Correlation analysis was used to reveal the correlation between the results of the systemic thinking assessment, professional skills exam, and course reflection index. The sign of the correlation coefficient (*γ*) represents positive or negative correlation, and the absolute value denotes the level of correlation. |*γ*|≤0.3, 0.3<|γ|≤0.7, and |*γ*|>0.7 represent weak, medium, and strong correlations, respectively. Table 6 lists the correlation coefficients between the three results.

**Table 6:** The correlation between the results of systemic thinking assessment, professional skill exam, and course reflection.

Pair	Systemic thinking assessment / Professional skills exam	Systemic thinking assessment / Course reflection index	Professional skills exam / Course reflection index
*γ*	0.461	0.503	0.261
Level	Medium	Medium	Weak

## 5 Discussion

### 5.1 How to build a community interaction open source learning framework to support engineering course teaching

Different from conventional teaching approaches, e.g., project-based, design-based, and problem-based learning, the core of the proposed framework laid in embedding community interaction elements in an open-source learning environment. The practice of the proposed learning framework included three stages: course preparation, theory lecture, and project practice. All stages had significant community interaction elements. The practical teaching template given in the course preparation suggested that the community experts should negotiate with students to determine the teaching forms. It encouraged the community team to integrate characteristic open-source resources based on fixed teaching links. The two-way selection mechanism between the community and students responded to students’ learning interests and balanced the guidance burdens of various community teams.

Theory lectures retained general open-source knowledge and simulation theory and discussed technical difficulties in the simulation procedure. It built a complete knowledge structure for students and a theoretical reserve for overcoming challenges with the practical guidance of the community teams. In project practice, the professional teachers provided teaching templates to community teams. It stipulated fixed links and suggested optional solutions, thereby reconciling the differences in the guidance of teams. Moreover, the two-way feedback mechanism was embedded to establish a smooth communication bridge between experts and students. It bridged the communication gap in interdisciplinary collaboration.

Therefore, the community interaction open-source learning framework can be seen as an improvement rather than a subversion of engineering courses. This study integrates the community interaction elements into the open-source learning environment for promoting individual sustainability development. It provides an example of engineering education for responding to the sustainability needs of open-source learning stakeholders.

### 5.2 How do participants view incorporating open-source learning framework in engineering course

The course reflection presented participants’ experiences, needs, and expectations of open-source learning. Regarding teaching materials, 81.7% of participants reported an interest in engineering simulation solutions, indicating their desire to learn how to apply finite element simulation theory to solve real-world challenges. 65.4% of participants mentioned the structured demonstration of the simulation procedure, and 69.5% of participants reported the benefit of the tutorial for abstract concept understanding. It demonstrates that the students consider the open-source platform as a valuable tool for structural performance analysis. The students expected to solve real challenges and viewed open-source simulation technology as a promising tool, implying participants’ positive attitudes towards incorporating the open-source learning frameworks into engineering courses.

Regarding curriculum setting, 85.8% of participants mentioned the benefits of the project practice, which is 2.45 times that of the theory lecture (34.9%). The possible reason is that the two-way feedback mechanism was embedded in the project practice, and the interaction elements of the first two stages also contributed to the project practice effectiveness. The two-way feedback mechanism created an indirect connection between experts and students. The professional teachers acted as facilitators of interdisciplinary collaboration, eliminating communication barriers caused by unfamiliarity between experts and students.

As the internal resistance of interdisciplinary collaboration decreased, the students developed a strong inner drive for open-source learning. Moreover, the teaching suggestions and two-way selection mechanism from the course preparation laid a foundation for interaction and enhanced students’ learning interest. The systematization and pertinence of theory lectures reduced the difficulty of solving problems for students and the guidance burden of experts in project practice. The interaction elements were possible triggers to enhance participants’ motivation to learn. Regarding the learning environment, 67.4% of participants reported a sense of achievement in contributing to the open-source community, indicating students’ desire to participate in open-source project development in the future.

### 5.3 How does the open-source learning framework strengthen participants’ sustainability competencies

The systemic thinking assessment results suggested that the open-source learning framework significantly enhanced participants’ systemic thinking. Compared with the pre-assessment of all participants, the mean score of the post-assessment improved by 22.3%. The results of the mechanical and electrical experimental groups were similar to those of all participants, excluding the possible errors introduced by differences in engineering majors. The professional skill exam findings showed that the experimental group had an overall pass rate advantage of 65.0% and 55.5% compared to the two control groups. The overall superiority of the experimental group was supported by formative results on pass rates of practical and theoretical exams. Therefore, the open-source learning framework explicitly enhances participants’ sustainability competencies in systemic thinking and professional skills and implies the promotion of interdisciplinary cooperation. Furthermore, the results validate the conclusions of previous literature, i.e., systemic thinking, professional skills, and interdisciplinary collaboration complement each other and form individual competencies in sustainability [37, 38].

The course reflection showed that 96.1% of participants reported their improvements. The participants’ improvements came from the teaching materials, course setting, and learning environment within the open-source learning framework. In teaching materials, 65.4% of participants mentioned that the improvements were related to the structured simulation procedure, guiding them to apply the simulation theory to the structural performance analysis. 69.5% of participants mentioned the tutorial’s benefit to conceptual understanding, indicating that the tutorial helped participants build prior knowledge of open-source project practices. The online courses and tutorials provided the scaffolding for participants to practice open-source projects. Besides, the open-source simulation platform provided participants with the simulation tool for project practice, showing the mapping relationship between design solutions and structural performance. Regarding course setting, 34.9% of participants believed that the theory lecture contributed significantly to improvement, and 85.8% of participants reported a strong correlation between project practice and improvements. It showed that the effectiveness of open-source learning frameworks for improving individual abilities grew rapidly throughout the theory lecture to project practice. The two-way feedback mechanism fostered interdisciplinary collaboration between students and experts, improving in simulation skills. In terms of the learning environment, 76.5% of participants believed that peer support in the open-source community was helpful for improvements. When the participants encounter challenges, they can seek peer help in the community. The open-source learning environment with immediate feedback helped to increase participants’ sense of engagement and gain, enabling active learning. The finding was supported by 67.4% of participants reported a sense of achievement. Also, the results confirm the conclusions of the existing literature [39, 40].

To sum up, the performance of the open-source learning framework was exhibited from three aspects: systemic thinking assessment, professional skill exam, and course reflection. The results of the three aspects show varying levels of correlation, as listed in Table 6. It indicated that the three measurement results supported each other, thereby enhancing the reliability of the finding that the proposed framework can strengthen participants’ sustainability competencies.

## 6 Limitations

One limitation is that open-source learning frameworks are difficult to extend directly into engineering courses without a practice module, because the course chosen for the experiment included theoretical and practice modules. The lack of practice modules will make it difficult to embed the element of community interactions. Thus, there might be applicability obstacles in teaching arrangements.

Another limitation may lie in the self-reported systemic thinking assessment. Several systemic thinking scales have been developed, and one of them was employed in this study. Using another scale may yield different results. Besides, the memory effect of participants on the pre-assessment may affect the self-reported results in the post-assessment. Therefore, the approach of systemic thinking assessment needs further improvement.

## 7 Conclusion

This empirical research proposes and tests a community interaction open-source learning framework. The framework creates an efficient open-source learning environment for professional courses to strengthen the engineering students’ sustainability competence. The contribution is to provide empirical evidence for incorporating community interaction elements into open-source learning. It responds to the needs of students, teachers, universities, open-source communities and enterprises for individual sustainability competence development. The proposed framework follows a general paradigm of competence development, focusing on teaching materials, course setting, and learning environment. In the framework practice, the embedded community interaction elements include community/student two-way choice, theory lecture and difficulty discussion, expert/student consultation on teaching forms, teacher/expert coordination on contents, and expert/student two-way feedback on schedules. The interaction elements between students, teachers, and experts enhance the effectiveness of open-source learning in engineering courses.

The findings suggest the following conclusions. Firstly, the participants welcome incorporating the community interaction open-source learning framework in engineering courses. They were interested in open-source project development and obtained a sense of achievement. Secondly, the community interaction elements in the open-source learning framework catered to students’ interests, reduced the practical guidance difficulty, balanced the guidance burden of the community teams, and strengthened the two-way feedback between teaching and learning. Thus, it realized a long-term mechanism to meet the needs of stakeholders in open-source learning. Thirdly, the open-source learning framework significantly enhanced participants’ systemic thinking, conceptual understanding, professional skills and interdisciplinary collaboration. It elevates participants’ key sustainability competencies, laying the foundation for them to become expertise-based innovators and contributors to the open-source community. Briefly, the open-source learning framework provides a practical approach for embedding sustainability competence development into engineering courses. Besides, sustainability competence is essential for non-engineering students. Extending open-source learning to this field and proposing advanced teaching approaches are our future research directions.

## Supporting information

S1 Data (XLS)

## References

[1] Bürgener L, Barth M. Sustainability competencies in teacher education: Making teacher education count in everyday school practice. J Clean Prod. 2018; 174: 821–826. 10.1016/j.jclepro.2017.10.263

[2] Wesselink R, Blok V, Van Leur S, Lans T, Dentoni D. Individual competencies for managers engaged in corporate sustainable management practices. J Clean Prod. 2015; 106: 497–506. 10.1016/j.jclepro.2014.10.093

[3] Busta L, Russo SE. Enhancing interdisciplinary and systems thinking with an integrative plant chemistry module applied in diverse undergraduate course settings. J. Chem Educ. 2020; 97(12): 4406–4413. 10.1021/acs.jchemed.0c00395

[4] Wiek A, Withycombe L, Redman CL. Key competencies in sustainability: a reference framework for academic program development. Sustain Sci. 2011; 6: 203–218. 10.1007/s11625-011-0132-6

[5] Asamoah MK. ICT officials’ opinion on deploying Open Source Learning Management System for teaching and learning in universities in a developing society. E-learning Digital M. 2021; 18(1): 18–38. 10.1177/2042753020946280

[6] Kortemeyer G, Kashy E, Benenson W, Bauer W. Experiences using the open-source learning content management and assessment system LON-CAPA in introductory physics courses. Am J Phys. 2008; 76(4): 438–444. 10.1119/1.2835046

[7] Cavus N, Zabadi T. A comparison of open source learning management systems. Procedia Soc Behav Sci. 2014; 143: 521–526. 10.1016/j.sbspro.2014.07.430

[8] Paton RM, Fluck AE, Scanlan JD. Engagement and retention in VET MOOCs and online courses: A systematic review of literature from 2013 to 2017. Comput Educ. 2018; 125: 191–201. 10.1016/j.compedu.2018.06.013

[9] Aksulu A, Wade MR. A comprehensive review and synthesis of open source research. J Assoc Inf Syst. 2010; 11(11): 576–656. 10.17705/1jais.00245

[10] Bossu C, Fountain W. Capacity-building in open education: an Australian approach. Open Prax. 2015;7(2): 123–132. https://www.learntechlib.org/p/151069/

[11] Sengers F, Wieczorek AJ, Raven R. Experimenting for sustainability transitions: A systematic literature review. Technol Forcast Soc. 2019; 145: 153–164. 10.1016/j.techfore.2016.08.031

[12] Kivimaa P, Boon W, Hyysalo S, Klerkx L. Towards a typology of intermediaries in sustainability transitions: A systematic review and a research agenda. Res Policy. 2019; 48(4): 1062–1075. 10.1016/j.respol.2018.10.006

[13] Lambrechts W, Gelderman CJ, Semeijn J, Verhoeven E. The role of individual sustainability competences in eco-design building projects. J Clean Prod. 2019; 208: 1631–1641. 10.1016/j.jclepro.2018.10.084

[14] Huang Z, Peng A, Yang T, Deng S, He Y. A design-based learning approach for fostering sustainability competency in engineering education. Sustainability. 2020; 12(7): 2958. 10.3390/su12072958

[15] Bielik T, Delen I, Krell M, Assaraf OBZ. Characterising the Literature on the Teaching and Learning of System Thinking and Complexity in STEM Education: a Bibliometric Analysis and Research Synthesis. Int J STEM Educ. 2023; 6: 199–231. 10.1007/s41979-023-00087-9

[16] Godfrey P, Crick RD, Huang S. Systems thinking, systems design and learning power in engineering education. Int J Eng Educ, 2014; 30(1): 112–127. http://hdl.handle.net/10453/115834

[17] Moirano R, Sánchez MA, Štěpánek L. Creative interdisciplinary collaboration: A systematic literature review. Think Skill Creat. 2020; 35: 100626. 10.1016/j.tsc.2019.100626

[18] Salas E, Rosen MA, DiazGranados D. Expertise-based intuition and decision making in organizations. J Manage. 2010; 36(4): 941–973. 10.1177/0149206309350084

[19] Caswell T, Henson S, Jensen M, Wiley D. Open educational resources: Enabling universal education. Int Rev Res Open Dis. 2008;9(1): 1–11. 10.19173/irrodl.v9i1.469

[20] Sadeghi M. A shift from classroom to distance learning: Advantages and limitations. Int J Res Eng Educ. 2019;4(1): 80–88. 10.29252/ijree.4.1.80

[21] Cronin C. Openness and praxis: Exploring the use of open educational practices in higher education. Int Rev Res Open Dis. 2017; 18(5): 15–34. 10.19173/irrodl.v18i5.3096

[22] Al Abri M, Dabbagh N. Open educational resources: A literature review. J Mason Grad Res. 2018;6(1), 83–104. 10.13021/G8jmgr.v6i1.2386

[23] Heradio R, Chacon J, Vargas H, Galan D, Saenz J, De La Torre L, et al. Open-source hardware in education: A systematic mapping study. IEEE Access. 2018; 6: 72094–72103. 10.1109/ACCESS.2018.2881929

[24] Pearce JM. Quantifying the value of open source hardware development. Modern Economy. 2015; 6, 1–11. 10.4236/me.2015.61001

[25] Daniel S, Stewart K. Open source project success: Resource access, flow, and integration. J Strategic Inf Syst. 2016; 25(3): 159–176. 10.1016/j.jsis.2016.02.006

[26] Potkonjak V, Gardner M, Callaghan V, Mattila P, Guetl C, Petrović VM, et al. Virtual laboratories for education in science, technology, and engineering: A review. Comput Educ. 2016; 95: 309–327. 10.1016/j.compedu.2016.02.002

[27] Linåker J, Regnell B, Damian D. A method for analyzing stakeholders’ influence on an open source software ecosystem’s requirements engineering process. Requir Eng. 2020; 25: 115–130. 10.1007/s00766-019-00310-3

[28] Ahn JY, Edwin A. An e-learning model for teaching mathematics on an open source learning platform. Int Rev Res Open Dis. 2018; 19(5): 255–267. 10.19173/irrodl.v19i5.3733

[29] Glassman M, Kang MJ. Teaching and learning through open source educative processes. Teach Teach Educ. 2016; 60: 281–290. 10.1016/j.tate.2016.09.002

[30] Van Rooij SW. Adopting open-source software applications in US higher education: A cross-disciplinary review of the literature. Rev Educ Res. 2019; 79(2): 682–701. 10.3102/0034654308325691

[31] Moqri M, Mei X, Qiu L, Bandyopadhyay S. Effect of “following” on contributions to open source communities. J Manage Inform Syst. 2018; 35(4): 1188–1217. 10.1080/07421222.2018.1523605

[32] Reid DP, Burridge J, Lowe DB, Drysdale TD. Open-source remote laboratory experiments for controls engineering education. Int J Mech Eng Educ. 2020; 50(4): 828–848. 10.1177/03064190221081451

[33] Dolansky MA, Moore SM, Palmieri PA, Singh MK. Development and validation of the systems thinking scale. J Gen Intern Med. 2020; 35: 2314–2320. doi: 10.1007/s11606-020-05830-1 32342481 PMC7403244

[34] Goulet-Pelletier JC, Cousineau D. A review of effect sizes and their confidence intervals, Part I: The Cohen’s d family. Quant Meth Psychol, 2018; 14(4): 242–265. 10.20982/TQMP.14.4.P242

[35] Obilor EI, Amadi EC. Test for significance of Pearson’s correlation coefficient. Int J Innov Math Stat Energ Pol. 2018; 6(1): 11–23. https://seahipaj.org/journals-ci/mar-2018/IJIMSEP/full/IJIMSEP-M-2-2018.pdf

[36] Olson JD, McAllister C, Grinnell LD, Gehrke Walters K, Appunn F. Applying Constant Comparative Method with Multiple Investigators and Inter-Coder Reliability. Qual Rep. 2016; 21(1): 26–42. https://core.ac.uk/download/pdf/51087586.pdf

[37] Van Laar E, Van Deursen AJ, Van Dijk JA, de Haan J. Determinants of 21st-century skills and 21st-century digital skills for workers: A systematic literature review. Sage Open. 2020;10(1), 1–14. 10.1177/2158244019900176

[38] Iqbal AM, Khan AS, Abdullah J, Kulathuramaiyer N, Senin AA. Blended system thinking approach to strengthen the education and training in university-industry research collaboration. Technol Anal Strateg. 2022; 34(4): 447–460. 10.1080/09537325.2021.1905790

[39] Tlili A, Huang R, Chang TW, Nascimbeni F, Burgos D. Open educational resources and practices in China: A systematic literature review. Sustainability. 2019; 11(18): 4867. 10.3390/su11184867

[40] Chen CM, Li MC, Chang WC, Chen XX. Developing a topic analysis instant feedback system to facilitate asynchronous online discussion effectiveness. Comput Educ. 2021; 163: 104095. 10.1016/j.compedu.2020.10409

